# Rapid Detection of Attomolar SARS-CoV-2 Nucleic Acids in All-Dielectric Metasurface Biosensors

**DOI:** 10.3390/bios12110987

**Published:** 2022-11-08

**Authors:** Masanobu Iwanaga

**Affiliations:** Research Center of Functional Materials, National Institute for Materials Science (NIMS), 1-1 Namiki, Tsukuba 305-0044, Japan; iwanaga.masanobu@nims.go.jp

**Keywords:** metasurface, fluorescence biosensor, SARS-CoV-2, complementary DNA, enhanced PCR, LAMP, rapid detection, attomolar detection

## Abstract

Worldwide infection due to SARS-CoV-2 revealed that short-time and extremely high-sensitivity detection of nucleic acids is a crucial technique for human beings. Polymerase chain reactions have been mainly used for the SARS-CoV-2 detection over the years. However, an advancement in quantification of the detection and shortening runtime is important for present and future use. Here, we report a rapid detection scheme that is a combination of nucleic acid amplification and a highly efficient fluorescence biosensor, that is, a metasurface biosensor composed of a pair of an all-dielectric metasurface and a microfluidic transparent chip. In the present scheme, we show a series of proof-of-concept experimental results that the metasurface biosensors detected amplicons originating from attomolar SARS-CoV-2 nucleic acids and that the amplification was implemented within 1 h. Furthermore, this detection capability substantially satisfies an official requirement of 100 RNA copies/140 μL, which is a criterion for the reliable infection tests.

## 1. Introduction

Since the invention of polymerase chain reaction (PCR) [1], how to reduce loads in nucleic amplification and how to make the amplification more sensitive have been long-term issues. Currently, quantitative PCR (qPCR) [2] (or real-time PCR) is the standard nucleic acid amplification technique that is commercially available. To obtain a higher sensitivity than that of qPCR, digital PCR (dPCR) [3,4,5], which is based on elaborate statistical analysis for attaining high sensitivity, is sometimes used at the expense of a higher cost as compared with that of qPCR. In the PCR techniques, fluorescence (FL) detection relies on conventional optical elements such as lens, mirror, filter, and photodetector.

Since 2019, the world has been deeply affected by the pandemic of SARS-CoV-2. The virus has genetic information in RNA; therefore, an infection test of high sensitivity requires the RNA detection, which can be conducted using reverse-transcription (RT) PCR. It is officially noticed that SARS-CoV-2 RT-PCR detection techniques are required to detect 100 copies/140 μL or 1.19 attomolar (aM) within 1 h amplification time [6]. RT-qPCR is a widely used standard method; the limit of detection (LOD) of RT-qPCR was reported to be located at 30–50 copies/test with the implementation of 40 cycles [7,8]; evaluating the LOD in the units of aM, it was 9.49–16.6 aM. Thus, the aforementioned official requirement is quite a high standard. The huge impact due to SARS-CoV-2 resulted in other numerous studies to detect it. There have been reports on rapid detection methods that were claimed to be competitive to qPCR or better than it: for example, dPCR using reaction droplets [9]; CRISPR-based detection in evanescent FL measurement [10]; and microfluidic (MF) chips using designed DNA probes and FL molecules [11]. To our knowledge, there has been no report that explicitly showed the superiority of a method over qPCR in terms of sensitivity.

In addition to the PCR techniques, loop-mediated isothermal amplification (LAMP), known as a nucleic acid amplification technique [12,13], is considered to have a better specificity than the PCR techniques with four primers, because LAMP employs six primers. However, a standard LAMP protocol for complementary DNA (cDNA) of SARS-CoV-2 was not sufficient in terms of sensitivity; thus, an initial-PCR-merged RT-LAMP was recently proposed, shortening the runtime to 35–40 min [14]. Nevertheless, the lowest detected target concentration was 2.77 aM, being 2.33-fold larger than the official requirement mentioned earlier. Colorimetric RT-LAMP using magnetic beads was reported, and the LOD for a 50 min runtime was 200 copies/mL, i.e., 0.33 aM [15]; however, the positive and negative results of the amplification were 50% at the claimed LOD. Therefore, in a normal sense, the reliable target concentrations were limited to ≥3.3 aM.

Here, we report proof-of-concept experiments for a new, highly sensitive, and short-time detection scheme for SARS-CoV-2 nucleic acids and show a series of the results indicating that aM detection was achieved in a short amplification time of <1 h. The results were obtained by incorporating modified-primer PCR and all-dielectric metasurfaces that have a feature in extremely sensitive FL detection [16].

**Figure 1:**
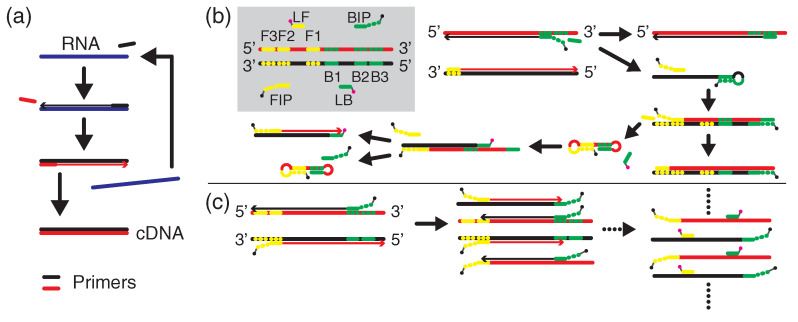
Nucleic acid transcription and amplifications. (**a**) Schematic of a cycle of reverse transcription from the original RNA (blue) to double-strand (ds) complementary DNA (cDNA) (red). Elongations thanks to polymerase reactions are indicated with arrows. (**b**) LAMP primers of F3, Forward inner primer (FIP), loop forward (LF) primer, B3, backward inner primer (BIP), and loop backward (LB) primer are summarized on gray domain. Dotted-line regions are complementary to F1, F2, F3, B1, B2, and B3, respectively. The FIP and BIP have biotin label (black dot) at the 5′ end with an extra sequence of TTT, and the LF and LB have fluorescence (FL) label (magenta dot) together with an extra sequence TTT. Only the initial several steps in LAMP are illustrated. (**c**) One-step PCR associated with FL-probe hybridization. Biotin-conjugated primers, that is, FIP and BIP in (**b**) contribute to amplification of the target cDNA (left to center). After the amplification, FL-labeled probes, that is, LF and LB in (**b**), are hybridized to the PCR amplicons (right).

Figure 1 illustrates the nucleic acid reactions related to this study. Figure 1a shows an RT cycle for an RNA target (blue), which is transcribed to DNA sequence using a primer. The complementary sequence (black) can hybridize the other primer (red) and results in double-strand (ds) cDNA. This cycle can be repeated, yielding the cDNA in a reproductive manner. Such an RT cycle can be conducted for 8–10 min [14] with the help of SD DNA polymerase [17]. It is therefore reasonable to assume that more than 10-fold cDNA is obtained in comparison with the original RNA. Thus, cDNA has been usually set as target in the PCR and LAMP detections.

In this study, we assumed that our detection started with the ds cDNA that was reproduced by 10-fold from the SARS-CoV-2 RNA. We examined two amplification methods of LAMP in Figure 1b and PCR in Figure 1c. The PCR and LAMP were one-step processes from the amplification to the FL-probe hybridization. We also incorporated the efficient FL-detection biosensors, that is, the all-dielectric metasurface biosensors [18,19]. In this comprehensive study, we reached an extremely low target concentration of 6 aM within 1 h amplification time, which strongly suggests that this scheme is capable of realizing sub-aM SARS-CoV-2 RNA detection.

## 2. Materials and Methods

### 2.1. DNA Sequence

Table 1 lists sequences of target and primers used in this study. The target was chosen from the sequence of SARS-CoV-2 BA.5.1, so-called *omicron* [20]; 360 bases from the positions 29,041 to 29,400 were taken from the full sequence and set as the target. The target sequence was not included in the initial SARS-CoV-2 found in Wuhan, China [21,22]. The 360-base ds cDNA was commercially produced as DNA fragment in an on-demand manner (Eurofins Genetics, Tokyo, Japan). We reconstituted the lyophilized cDNA in Table 1 with Tris-HCl 10 mM, which was prepared from 1 molar (M) Tris-HCl (314-90065, Nippon Gene, Tokyo, Japan) in dilution with diethylpyrocarbonate (DEPC)-treated water (318-90203, Nippon Gene). The target cDNA was reconstituted at 1 μM and diluted to an aM range. The dilution was very extensive, approximately $10^{9}$-fold; therefore, we intended to stabilize the extremely diluted target, so we prepared a buffer for dilution, Tris-HCl 10 mM with 10 nM oligonucleotide, which comprised CGTACCATGCATGCATGTTT and did not hybridize with the cDNA and primers.

The primer design for LAMP was implemented using a web-based software, Primer-Explorer V5 [23], as listed in Table 1. As illustrated in Figure 1b, F3 and B3 primers were located near the 5𠌩 ends in the target cDNA. Other F2, F1, B2, and B1 sequences were also determined. Forward inner primer (FIP) was a combined sequence of F2 and F1c, where F1c denotes complementary to F1. Similarly, backward inner primer (BIP) was defined as B2-B1c, where B1c was complementary to B1. The six primers (F3, B3, FIP, BIP, LF, and LB) were produced through oligosynthesis and purified via high-performance liquid chromatography (Eurofins Genetics). The melting temperature $T_{m}$ was estimated using an empirical equation for guanine–cytosine (GC) percent in the single strands [24]:

(1) $$T_{m}=81.5+16.6\log_{10}\left[{\mathrm{Na}}^{+}\right]+0.41\times \mathrm{GC}\left(\%\right)-500/L$$

where *L* is the base length of the sequence and $\left[{\mathrm{Na}}^{+}\right]$ is Na ion concentration in the units of M. It is to be noted that the values of $T_{m}$ were evaluated assuming $[{\mathrm{Na}}^{+}]=50$ mM. From Equation (1), $T_{m}$ of F3, B3, FIP, BIP, LF, and LB were estimated to be 55, 50, 64, 66, 53, and 55 ${}^{\circ }$C, respectively.

**Figure 2:**
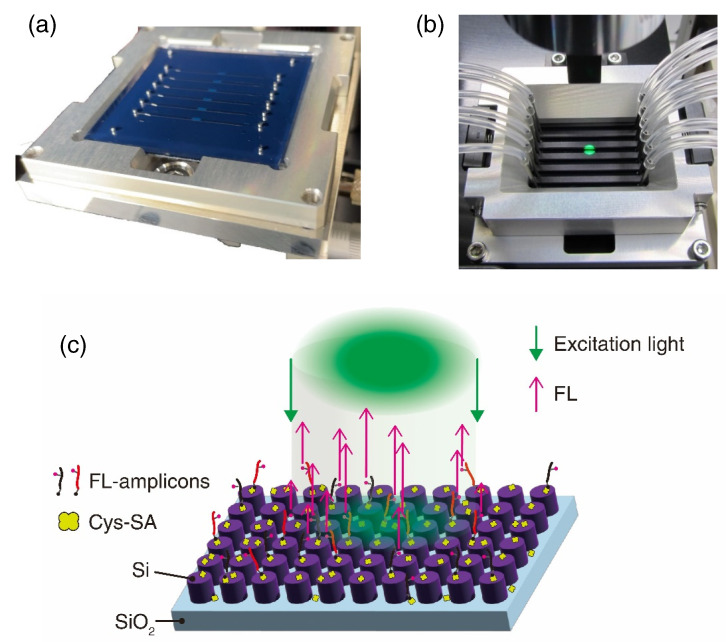
Metasurface biosensors. (**a**) Photograph. (**b**) The metasurface biosensor holder. Tubes are connected. Green spot is focused LED light for excitation. (**c**) Schematic of immobilized FL-labeled amplicons and FL emission on the all-dielectric metasurface.

In practice, the FIP and BIP sequences were conjugated with biotin at the 5′ end, together with a TTT sequence; that is, [Bio]TTT-FIP and [Bio]TTT-BIP were used in the amplification processes, as depicted in Figure 1b,c, where [Bio] denotes biotin. Owing to the biotin label, the amplicons were enabled to specifically absorb Cys-streptavidin (Cys-SA, PRO1005, ClickBiosystems, Richardson, TX, USA) that was immobilized on the outermost surface of Si nanorods forming the metasurface; the configuration is illustrated in Figure 2. For FL detection, FL probes are necessary. In this study, the LF and LB played the role. FL molecules named HEX [25] were conjugated at the 5′ end of the LF and LB together with an extra sequence TTT; symbolically, the FL probes were expressed as [HEX]TTT-LF and [HEX]TTT-LB. Note that the FL probes did not lose the function as primers because the 3′ end was free.

### 2.2. Nucleic Acid Amplification Procedures

Using the six primers addressed in Section 2.1, LAMP was implemented using a commercial kit (LAMP MASTER for Turbidity, Nippon Gene). One test was adjusted to 25 μL in total: 12.5 μL LAMP MATER mix including DNA polymerase and optimized buffers, 5.0 μL primer mix, 3.5 μL DEPC-treated water, and 4 μL target solution noted in Section 2.1. The amounts of F3, B3, FIP, BIP, LF, and LB in the test were 10, 10, 40, 40, 20, and 20 pmol, respectively. In the LAMP reactions, temperatures were first set to 60–70 ${}^{\circ }$C for 60 min and then down to 25 ${}^{\circ }$C for 5 min in a thermal cycler (Biometra TAdvanced 96SG, Analytik Jena, Gettingen, Germany). After the reaction, FL was measured in an automated system described later (Section 2.4).

PCR was conducted using a primer set of the FIP, BIP, LF, and LB, originally designed for LAMP in Table 1, and a commercial kit (TaKaRa Taq Hot Start Version, Takara-Bio, Kusatsu, Japan). Although PCR primers usually consist of 18–22 bases, we used the modified primers, FIP and BIP, for the PCR. A benefit of using the modified primers was their higher annealing temperatures compared with those of ordinary 20-base primers, which could reduce false reactions coming from accidental annealing to the target. One PCR test was adjusted to 50 μL: 5 μL PCR mix including optimized buffers, 4 μL dNTP mixture, 0.25 μL Taq HotStart, 3 μL primer mix, 32.75 μL DEPC-treated water, and 5 μL target solution in Section 2.1. The amount of primers, FIP and BIP, was set to 40 pmol in common, and that of FL probes, LF and LB, was 20 pmol. Temperature condition for the PCR was set in the thermal cycler as follows: 98 ${}^{\circ }$C for 10 s, (60 ${}^{\circ }$C for 30 s → 70 ${}^{\circ }$C for 30 s → 95 ${}^{\circ }$C for 5 s) × 35 cycles, 50 ${}^{\circ }$C for 15 min, 25 ${}^{\circ }$C for 5 min. The amplification runtime from the initiation to the end of thermal cycling was approximately 54 min, taking account of the temperature lifting time and being <1 h. A step keeping at 50 ${}^{\circ }$C for 15 min was included to make sure the hybridization of the FL probes (the LF and LB) with the PCR amplicons. After the series of temperature controls, the sample was readily measured in the automated FL-detection system (Section 2.4).

### 2.3. Preparation of All-Dielectric Metasurface Biosensors

The all-dielectric metasurface biosensor consisted of a self-absorbed pair of a metasurface substrate of 45×45 mm${}^{2}$ and a polydimethylsiloxane (PDMS) MF chip. A photograph of a metasurface biosensor is shown in Figure 2a. The PDMS chip was transparent, and had inlet and outlet holes for six MF channels. The metasurface biosensor was set in a holder, as seen in Figure 2a,b, and was connected to tubes for liquid flow. FL was measured in the configuration.

The all-dielectric metasurface consisted of periodic Si-nanorod array, as illustrated in Figure 2c. The Si-nanorod array has a series of prominent electromagnetic resonances, which were observed in high-contrast reflectance spectrum [16]; a higher magnetic resonance was found to contribute to significant (∼1000-fold) FL-intensity enhancement. The design involved the following: a periodicity of 300 nm, a circular diameter of 220 nm, and a height of 200 nm. We went through nanolithography from electron-beam drawing of dry etching of Si on insulator and prepared the metasurface substrates that were almost faithful to the design. The detailed procedures were described in the previous publications [18,19].

### 2.4. Automated MF Procedures

MF procedures were automated in the following motions: flow of liquid reagents in time and rate, change in the liquid reagents, FL imaging, and the image collection. To realize the motions, three electric-driven stages were controlled by a software that unifies all the motions. Quantitative flow of the liquid reagents was conducted with a small rotary pump (RP-6R01S-3P6A-DC10VS, Takasago Fluidic Systems, Nagoya, Japan), which controlled six-channel flow at the same time.

After the LAMP or PCR, the test liquid samples were flowed in the metasurface biosensors. MF flow protocol was as follows. First, phosphate-buffered saline (PBS, 164-25511, Fujifilm Wako Pure Chemical, Osaka, Japan) of pH 7.4 was flowed at 75–80 μL/min for 3 min in the MF channels and filled the MF paths with the buffer. Second, Cys-SA diluted to 2 μg/mL with 98% PBS and 2% glycerin (070-04941, Fujifilm Wako Pure Chemical) was flowed on the metasurfaces at 10–11 μL/min for 14 min. PBS rinse was conducted at 10–11 μL/min for 6 min. Then, background measurement for FL detection was implemented. After that, the test liquid samples that adjusted to 100 μL with the PBS were flowed at 9.5±0.5 μL/min for 10 min. Succeedingly, PBS rinse was conducted at 18–20 μL/min for 5 min. At the end, FL measurement was executed for 2 s exposure under green LED excitation. All the procedures were set in advance on a controlling computer and were automatically implemented. The core FL detection time was the time from the flow of the test liquid to the final PBS rinse and 15 min (including the auto-FL measurement), because the flow before the test liquid can be conducted independently of the nucleic acid amplification. Thus, the FL detection using the metasurface biosensors was implemented in a short time.

### 2.5. FL Measurement

An optical unit to conduct FL measurement on the metasurface biosensors was incorporated in the automated MF system in Section 2.4. A 10× objective lens of numerical aperture (NA) 0.28 (M Plan Apo, Mitsutoyo, Kawasaki, Japan) focused the illumination green LED (M530F2, Thorlabs, Newton, NJ, USA) light and collected the FL emitted on the metasurface. An uncooled CCD camera (Infinity-3S, Teledyne-Lumenera, Ottawa, ON, Canada) was used in the automated MF system; in the FL and background measurement, exposure time was set to 2 s and gain of signal was set to 10. Without any active cooling mechanism, the background was not low. When evaluating the measured FL intensities, we subtracted the background level in data analysis.

To conduct low-background FL measurement for the extremely diluted samples, confocal FL microscope (Stellaris 5, Leica, Wetzlar, Germany) was used. In the photon counting mode, the background coming from the instrument was suppressed to almost zero. A 10× objective lens of NA 0.32 (HC PL FLUOTAR, Leica) was used. In accordance with the FL-molecule HEX, excitation wavelength was set to 521 nm and detection wavelengths were set to 570–700 nm. An FL image was acquired through 10-frame accumulation.

## 3. Results

### 3.1. FL Detection via LAMP

**Figure 3:**
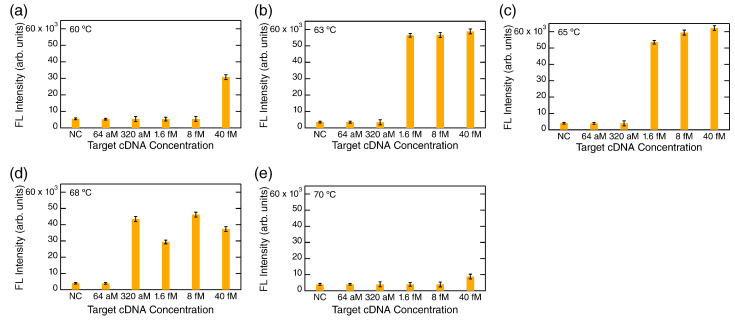
FL detection after LAMP at various reaction temperatures: (**a**) 60 ${}^{\circ }$C. (**b**) 63 ${}^{\circ }$C. (**c**) 65 ${}^{\circ }$C. (**d**) 68 ${}^{\circ }$C. (**e**) 70 ${}^{\circ }$C. The reaction time was fixed at 60 min in these five conditions. Measured FL intensities are shown with error bars. NC denotes negative control, meaning 0 molar (M) target in the Tris-HCl buffer for dilution.

Figure 3 shows a typical measured data set of FL detection at the end point of LAMP for the SARS-CoV-2 cDNA. The FL intensities were acquired in the automation MF system, shown as yellow bars together with error bars. Target concentrations were varied from 40 femtomolar (fM) to 64 aM. Negative control (NC), which was 0 M target concentration, was also measured. The amplification reaction temperatures were set to 60, 63, 65, 68, and 70 ${}^{\circ }$C in Figure 3a–e, respectively.

Prominent FL intensities were observed at 63–68 ${}^{\circ }$C, whereas the FL intensities at 60 and 70 ${}^{\circ }$C were suppressed, suggesting that 60 and 70 ${}^{\circ }$C are out of an optimal temperature range of the DNA polymerase. At 63 and 65 ${}^{\circ }$C, LAMP was probably saturated for the concentrations ≥1.6 fM; in contrast, LAMP did not take place at the concentrations ≤320 aM, and the FL intensities were almost the same level to the NC, indicating that there was no FL signal. The results were difficult to understand because the concentration difference between 1.6 fM and 320 aM is only 5-fold; therefore, we considered some signature of LAMP to be observable for 320 aM when the 1.6 fM target exhibited LAMP saturation. At 68 ${}^{\circ }$C, the FL signals coming from LAMP were observed even at 320 aM, while the FL intensities did not reach a level of saturation and suggested suppression of LAMP reaction by 20–50% in comparison with the results at 63 and 65 ${}^{\circ }$C. The results at 68 ${}^{\circ }$C imply that LAMP reaction became unstable compared with the reactions at 63 and 65 ${}^{\circ }$C. Overall, the results in Figure 3 were similar to the result in the previous report, showing that the lowest detected concentration was 277 aM in the quantitative LAMP [14].

### 3.2. FL-Enhanced Detection via PCR

**Figure 4:**
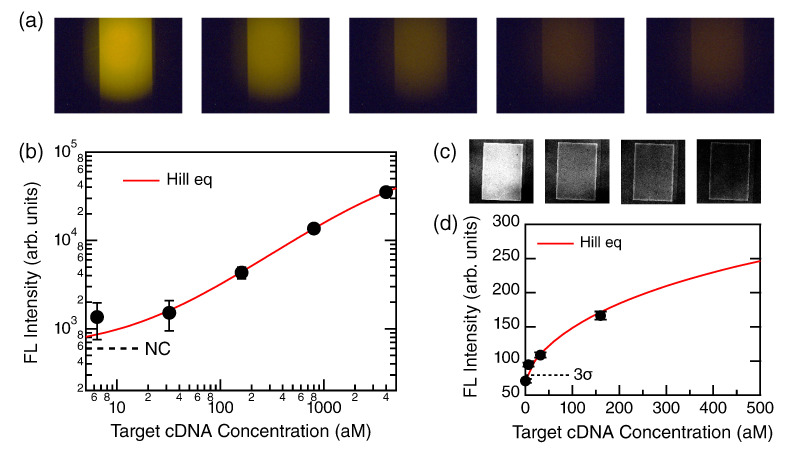
PCR results. (**a**) FL images at target concentrations of 4000 aM, 800 aM, 160 aM, 32 aM, and 6.4 aM, shown from left to right, respectively. The images were taken in the automated system (Section 2.4). (**b**) Log–log plot of FL intensity (black closed circle with error bar) for the target concentrations. The data were fitted using the Hill equation (red, Equation (2)). Dashed line indicates the NC (or 0 M) level. (**c**) Confocal FL images: target concentrations at 160 aM, 32 aM, 6.4 aM, and 0 M from left to right, respectively. Each image is a square shape of 1.1 mm side. (**d**) A magnified view of the confocal FL-intensity plot near 0 M, which is presented in a linear manner. Fitted curve using the Hill equation is shown with red. The 3σ level from the background level at 0 M is shown with dotted line.

Figure 4a shows a set of measured FL images at the end point of PCR. The five images displayed left to right correspond to target concentrations of 4000 aM, 800 aM, 160 aM, 32 aM, and 6.4 aM, respectively. Distinctly bright areas were the metasurface. The color exhibits raw color. We mention that the brightness was increased by 40% and the contrast was decreased by −40% for better visibility.

The profile of FL intensity in Figure 4b is approximated by Hill equation [26] because binding of the biotin-labeled amplicons to Cys-SA is a chemical reaction under equilibrium in the MF channels. The Hill equation is mathematically equivalent to the so-called four-parameter logistic equation [18]:

(2) $$y=y_{0}+\left(S-y_{0}\right)\frac{x^{n}}{x^{n}+K_{D}^{n}}$$

where *y* denotes the FL intensity, $y_{0}$ the background level without any target, *S* the saturation signal intensity, which was regarded as a proportional constant in fitting, *x* the concentration of target, *n* the degree of cooperative reaction, and $K_{D}$ dissociation constant [27,28]. The parameter *n* means an anti-cooperative binding reaction for n<1 and a cooperative reaction for n≥1 [29]. In fitting the data in Figure 3b, the baseline $y_{0}$ was set to be the NC level, and the variables were *n*, $K_{D}$, and *S*. By fitting, we found that n=0.84, $K_{D}=5823$ aM, and S=82,728. The result suggests that the binding reaction is anti-cooperative, which was often observed between biotin and streptavidin [18,30].

To examine the FL signals at the low concentrations, it was necessary to conduct low-background FL measurement; thus, we used confocal FL microscopy. Figure 4c presents the confocal FL images at the target concentrations of 160 aM, 32 aM, 6.4 aM, and 0 M, which are displayed from left to right. The metasurface biosensor was the same as that in Figure 4a. Qualitatively, it was confirmed that the higher target concentration was observed as relatively brighter images and that the metasurface flowed with the 0 M reaction sample was the darkest among the four images. It is to be noted that the four images are presented in a common setting on the brightness and contrast.

The FL intensities in Figure 4c were quantified and plotted in Figure 4d (closed black circles with error bars), shown in a linear representation. From the *x* value at the cross-point of the fitted Hill curve (red) and 3σ (σ: standard deviation) dotted line from the 0 M level in Figure 4d, LOD was numerically found to be 5.86 aM, which was slightly smaller than the measured concentration of 6.4 aM.

## 4. Discussion

We conducted the experiments for SARS-CoV-2 cDNA detection. As a result, the target was experimentally detected at 6.4 aM, and the LOD was found to be 5.86 aM from the analysis in Section 3.2. Thus, we demonstrated that the present detection scheme combining primer-modified PCR with the all-dielectric metasurface biosensors reached a very low target concentration of approximately 6 aM. It was assumed that the cDNA was produced via a reproductive RT process and that the original RNA was increased by 10-fold. Therefore, this scheme is reasonably considered to reach an LOD of 0.6 aM for the SARS-CoV-2 RNA, which is a half of the official requirement for an LOD of 1.19 aM [6].

Here, we compare the present results with other results reported to date in Table 2. In the report [7], RT-qPCR was conducted for the clinical samples. Going through the 40-cycle PCR, the LOD of 9.49–16.6 aM was obtained [7]. In contrast, this study conducted a 35-cycle PCR and reached an LOD of 5.86 aM for the cDNA target. When we focus only on the PCR cycles and do not take the reproduction in the RT process into account, the scheme in this study can provide $2^{5}$(=32)-fold efficiency at the maximum in terms of sensitivity compared with the RT-qPCR result. In addition, the LOD is approximately 2-fold better than that in the report in [7]. In total, 64-fold detection efficiency is attained for the qPCR. Such a substantial advance was realized by incorporating the all-dielectric metasurface biosensors.

As a fast PCR trial, PCR1100 was reported [31]. The amplification was 20 min. The LOD was claimed to be 1.7–5.3 aM; however, it is to be pointed out that detection ratio at the LOD was at a range from 3/6 (that is, 3 positive samples in total 6 tests) to 1/6, respectively. From the result, it is unclear how the LOD was quantitatively identified from the detection ratio of 50% or less.

LAMP is generally considered to be superior to PCR in terms of specificity and inferior in terms of sensitivity. Regarding SARS-CoV-2 RNA detection, it was reported that the quantitative LAMP showed an LOD of 277 aM or worse [14,32,33]. In this study, we observed LAMP signals at 320 aM, whereas it was difficult to determine the LOD because the signals showed saturation and sudden disappearance of the FL signals, as shown in Figure 3. Similar behaviors in LAMP were reported at a low target concentration range [34,35], where stochastic positive responses were observed; for example, the target at 17 aM was detected at a ratio of 3/4, and the target at 1.7 aM was detected at a ratio of 1/4 [34]. Generally, such stochastic results are unreliable in a situation that infection tests require definite evidences to judge positive or negative. Thus, it is likely that LAMP is difficult at present to be handled as a quantitative method at extremely low target concentration ranges.

We set a target that comprises a short cDNA fragment of 360 bases. The RT of RNA usually sets the target region in a part of the total RNA. Therefore, our target setting is a rational choice. Similar detections are also possible for influenza that has an approximately 5000-base RNA in total and 8 domains fewer than 800 bases. Multi-target infection tests will be designed based on the present scheme of PCR + metasurface sensors.

## 5. Conclusions

We conducted a series of proof-of-concept experiments for high-sensitivity detection of the SARS-CoV-2 nucleic acids. When we set the detection target to be cDNA, the scheme that combines the primer-modified PCR with the all-dielectric metasurface biosensors succeeded in the 6 aM target detection. The amplification time was <1 h. The FL detection in the metasurface biosensors was implemented in the automated manner; the substantial runtime for FL detection was 15 min. We assumed reasonably that an RT-reproduction of the RNA yielded 10-fold cDNA and evaluated the LOD for the RNA to be 0.6 aM. Consequently, it is concluded that the present detection scheme can meet the official requirement for the RNA LOD of 1.19 aM.

## Figures and Tables

**Table 1:** Sequences information: a target of 360 bases was taken from the sequence of SARS-CoV-2 BA.5.1. One of the ds cDNA (red line in Figure 1b) is listed here. Six primers of F3, B3, FIP, BIP, LF, and LB were designed for LAMP reaction. F3, B3, FIP, BIP, LF, and LB have 18, 18, 43, 42, 19, and 19 bases, respectively. Conjugations to some of the primers are described in the text.

Role	Sequence, Shown from 5‣- to 3‣-End
Target	AAGCCTTACCGCAGAGACAGAAGAAACAGCAAACTGTGACTCTTC
	TTCCTGCTGCAGATTTGGATGATTTCTCCAAACAATTGCAACAATCC
	ATGAGCCGTGCTGACTCAACTCAGGCCTAAACTCATGCAGACCACA
	CAAGGCAGATGGGCTATATAAACGTTTTCGCTTTTCCGTTTACGATAT
	ATAGTCTACTCTTGTGCAGAATGAATTCTCGTAACTACATAGCACAA
	GTAGATGTAGTTAACTTTAATTTCACATAGCAATCTTTAATCAGTGTG
	TAACATTAGGGAGGACTTGAAAGAGCCACCACATTTTCACCTACAG
	TGAACAATGCTAGGGAGAGCTGCCTATATGGAA
F3	CCTTACCGCAGAGACAGA
B3	TCGTAAACGGAAAAGCGA
FIP	TGCAATTGTTTGGAGAAATCATCCAGAAACAGCAAACTGTGAC
BIP	ACAATCCATGAGCCGTGCTGAAACGTTTATATAGCCCATCTG
LF	AATCTGCAGCAGGAAGAAG
LB	CTCAGGCCTAAACTCATGC

**Table 2:** SARS-CoV-2 detection for nucleic acids. MSF denotes the all-dielectric metasurface sensor is in this study. ${}^{†}$ Detection ratio of positive results discussed in the text was in a range from 3/6 to 1/6.

Target	Method	LOD	Amplified Condition
RNA	RT-qPCR [7]	9.49–16.6 aM	40 cycles
RNA	RT-qPCR [31]	1.7–5.3 aM ${}^{†}$	20 min, 50 cycles
RNA	RT-PCR + LAMP [14]	2.77 aM	35–40 min
cDNA	LAMP + MSF	320 aM	60 min
cDNA	PCR + MSF	5.86 aM	35 cycles

## Data Availability

Data in this article are available from the author upon reasonable request.

## Abbreviations

The following general abbreviations are used without definition in this article:

SARS-CoV-2	Severe acute respiratory syndrome coronavirus 2
DNA	Deoxyribonucleic acid
RNA	Ribonucleic acid
G	Guanine
C	Cytosine
T	Thymine
A	Adenine
LED	Light-emitting device
CCD	Charge-coupled device
